# Evolution of Online Health-Related Information Seeking in France From 2010 to 2017: Results From Nationally Representative Surveys

**DOI:** 10.2196/18799

**Published:** 2021-04-14

**Authors:** Pauline Ducrot, Ilaria Montagni, Viet Nguyen Thanh, Anne-Juliette Serry, Jean-Baptiste Richard

**Affiliations:** 1 Santé publique France, French national public health agency Saint-Maurice France; 2 Bordeaux Population Health Research Center, Team HEALTHY University of Bordeaux Inserm Bordeaux France

**Keywords:** internet, health information, information-seeking behavior, eHealth literacy

## Abstract

**Background:**

Given the rapid ongoing progression of the internet and increase in health information available from disparate online sources, it is important to understand how these changes impact online health information-seeking behavior of the population and the way of managing one’s health.

**Objective:**

This paper aims at describing the evolution of internet use as a source of health information between 2010 and 2017, as well as the characteristics of online health information seekers, topics of interest, sources of information, and trust in retrieved information and potential impact on behavior.

**Methods:**

Data from the French nationally representative surveys Health Barometers were used (N=4141 in 2010, 4811 in 2014, and 6255 in 2017). Evolutions over time were assessed using chi-square tests. Associations with sociodemographic characteristics and health status were evaluated using logistic regression models.

**Results:**

The use of the internet as a source of health information rose between 2010 and 2014 (from 37.3% to 67.9%, *P*<.001) but decreased significantly in 2017 (60.3%, *P*<.001). Overall, the profile of health information seekers compared with nonseekers did not change over time. They were more likely to be women, to be younger, to have a higher educational level, to have a higher household income, and to be executives. Between 2014 and 2017, the proportion of those who did not pay attention to the source of information significantly increased to reach 39.7% (*P*<.001). In 2017 as in 2014, general health-related websites remained the first source of information (38.6%) while institutional websites were the third source (8.1%). Most information seekers trusted the information found online in 2010 (more than 80%), with a slight decrease between 2014 and 2017 (*P*=.048). Among individual characteristics, trust in the information was the main determinant of the way of managing one’s health (odds ratio 4.06, 95% CI 3.26-5.06).

**Conclusions:**

After a rapid growth in the internet use for seeking health information in the 2010 to 2014 period, a decrease was recorded in 2017, in parallel with a decrease in trust in the quality and reliability of information found online. These findings underline the need for public health authorities to increase citizens’ eHealth literacy and to provide alternative trustworthy sources combining the popularity and accessibility of general health information websites.

## Introduction

In Europe, internet access is now democratized with 80% of the households using the internet for personal use [1]. Thus, over the last decade, the internet has become a major source of information including health-related information, with about 6 out of 10 Europeans reporting seeking health information online in the past year [1].

Using the internet as a health information source has many advantages. By offering quick, easy, timely, and low-cost access to the information, the internet tends to expand access to health messages [2], thus impacting citizens’ management of their health. Providing the information is reliable, citizens may increase their health knowledge, better understand the risks and benefits of some treatments, and participate in their health care decision making [3,4]. The internet also provides the possibility of personalized feedback [2,5] and contributes to addressing issues of geographical or mobility isolation and anonymity [2,5]. Indeed, the privacy offered by online information is particularly valuable for individuals searching for information on sensitive topics [2,5,6]. However, these advantages can easily turn into disadvantages, since the quality and authority of health-related information is debatable, and identifying trustworthy versus not trustworthy sites is challenging [2]. In fact, the multiplication of health-related websites increasingly raised the issue of accessibility to accurate and trustworthy information [2,7-9]. In light of this, users must have appropriate capacities to access, understand, appraise, and use health-related information in a digital environment (ie, eHealth literacy) [10]. Previous studies have shown that a high level of eHealth literacy is associated with good management of one’s health [11-13].

Online health information-seeking behavior depends on the characteristics of individual internet users, which might determine the reasons to search for health-related information online. Indeed, the literature has highlighted several predictors of online health information seeking, such as sociodemographic characteristics and overall health conditions [14]. Poor health, being female, being younger, having a diploma, and having higher income are associated with seeking health information online [15-19]. Similar trends were observed for eHealth literacy according to age and educational level. However, no significant difference has been reported between men and women [13,20].

A few studies have recently explored the trends in the use of the internet for health information seeking. The majority of them have been conducted in the United States [21-23] or have focused on specific population subgroups such as older people [24,25], pregnant women [26,27], children and adolescents [28-30], cancer survivors [31], or sexual minorities [32]. Two studies were conducted in Europe, in Finland [33] and Norway [34], but they considered data up to 2007 or 2009. In the European Union, eHealth literacy has been identified as a priority to address health inequalities in the eHealth Action Plan 2012-2020. The Eurobarometer on digital health literacy performed in 2014 supported this objective by assessing how Europeans use online information to help manage their own health. However, to our knowledge, no recent data exist on the evolution of such practice.

In addition, despite the rapid ongoing progression of the internet and overall perception of an increase in health information available from disparate online sources, the evolution of such sources used to seek information has been not documented so far. Finally, it is important to understand how these changes impact the online health information-seeking behavior of the population in order to offer appropriate solutions to disseminate reliable health messages.

The main objective of this article, therefore, was to describe, based on nationally representative surveys, the evolution of internet use as a source of health information between 2010 and 2017 in France. More specifically, this study was aimed at (1) describing the prevalence of internet use for health-related purposes and the characteristics of online health information seekers over time; (2) assessing the evolutions of topics of interest and sources of health-related information found online; and (3) investigating the attention paid to sources of information, the trust in retrieved information, and the potential impact on management of one’s health.

## Methods

### Survey Methodology and Participants

Data were extracted from 3 national surveys (called Health Barometers) conducted in 2010, 2014, and 2017 by the French national public health agency (Santé publique France, formerly the French Institute for Prevention and Health Education or INPES) in consultation with the French Ministry of Health [20]. Health Barometers are cross-sectional surveys of random representative samples of the French population conducted using computer-assisted telephone interviewing. These surveys were designed to measure the evolution of key indicators regarding health-related behaviors, attitudes, and opinions in the general population. The questionnaires and the data collection methods are available on the official survey website [35,36]. Briefly, Health Barometers evaluate different health topics such as addiction (tobacco, alcohol, illegal drugs), mental health, sexuality, nutrition, or vaccination, as well as use of the internet for health. The full questionnaire is designed not to exceed 30 minutes completion time. The general part of the questionnaire, lasting 20 minutes, is addressed to all participants, and specific parts, lasting 10 minutes, are asked to different subsamples.

Health Barometers are based on a 2-stage random sampling design: sampling of telephone numbers covering all metropolitan French regions and random selection of one member of the household, using the method proposed by Kish [37]. In 2010, because of the increasing rate of households that had abandoned their landline telephones for cell phones, a cell-only sample was added (12% of the sample to keep the same rate as in 2010 in France). In 2014, since a section of the population including people also having a landline preferred using a mobile phone, 2 overlapping samples were constituted: one surveyed by landline and the other by mobile phone [38].

For households of the landline sample, one person was randomly selected among eligible persons living in the household (aged 15 to 75 years in 2014 and 2017 and 15 to 85 years in 2010, speaking French). In the cellular sample, selection was done among persons sharing the cell telephone (when such sharing was reported).

If a household or individual refused to participate or could not be reached, they were not replaced in the study. For this reason, considerable efforts were made to reach households and increase the response rate: a formal request to participate explaining who was conducting the study and the goals of the survey was sent by electronic mail or letter to participant (when such information could be found using a national reverse directory). For every sampled number, up to 40 attempts were made to complete an interview. The calls were staggered over times of day and days of the week to maximize the chances of making contact with a potential respondent. When the selected individual was reached but unavailable, an appointment was made. Individuals unwilling to participate at first were contacted again by specialized interviewers in order to recruit them. Response rate of the 3 surveys was 53% in 2010, 61% in 2014, and 49% in 2017. All collected data were anonymous and self-reported. The survey was approved by the National Data Protection Authority and complied with the European Union General Data Protection Regulation.

### Data Collection

#### Sociodemographic and Economic Characteristics and Health Status

Participants were asked to provide sociodemographic data, including gender (men, women), age in categories (18-24 years, 25-34 years, 35-44 years, 45-54 years, 55-54 years, 65-75 years, >75 years), educational level (primary, secondary, postsecondary), employment status (working, student, unemployed, retired, other), occupational category (farmers, artisans, executives, intermediate profession, employees, manual workers) and monthly income. Occupational category was reported for the respondent (using the last job position for unemployed and retired people) or for the reference person in the household if the respondent had never worked before (ie, student). Monthly household income was calculated per consumption unit (CU), where one CU is attributed for the first adult in the household, 0.5 CU for other persons aged 14 years or older, and 0.3 CU for children under 14 years, following national statistics methodology and guidelines [39]. Income categories were defined using the tertiles of the entire database, including the 3 years of data sets. In addition, participants were asked if they have a chronic disease (yes/no).

#### Internet Use for Health Information Seeking

In 2010, 2014, and 2017, participants were asked whether they had used the internet to search for health-related information or advice in the last 12 months (yes/no/no access to the internet). This question was used to identify health information seekers versus non–health information seekers. Only online health seekers were further asked about the trust they had in health-related information obtained on the internet. The responses were rated on a 4-point Likert scale ranging from 1 (not trustworthy at all) to 4 (totally trustworthy) and grouped into 2 categories (not trustworthy vs trustworthy). In order to understand the effect of using the internet on the doctor/patient relationship, individuals were asked whether the information and advice found on the internet had changed the way they were taking care of their health (4-point Likert scale from not at all to definitely yes, further grouped in 2 categories, yes vs no). In addition, they were asked if their use of the internet led them to visit their doctor more often, less often, or to the same extent as they did before using the internet for health purposes.

In 2014 and 2017, online health information seekers were also asked about the topics of their searches including (1) general health and illnesses, medical news, and treatments; (2) sexually-related health risk; (3) contraception and methods to avoid pregnancy; (4) nutrition, weight gain, or eating disorders; (5) pregnancy or maternity; (6) child health and illness; (7) alcohol; (8) tobacco; (9) cannabis and other drugs; and (10) electronic cigarettes (the latter assessed in 2017 only).

In 2014 and 2017, the questionnaire also included questions about the source of health information in general (forum, health information website, or did not pay attention to the information source) and the specific websites used for searching health information. Spontaneous reponses of participants were then categorized into different types of websites including general health-related websites; Doctissimo (a popular French website dedicated to general health mentioned by name by a large number of participants); social media; Wikipedia; institutional websites, Websites from health professional, patient association, scientific database; and others.

In 2010, questions were asked to assess why some people did not seek online health information (sufficiently informed, not interested in such information, better to ask these questions to a doctor, distrust in retrieved online information, do not think about it). Online health information seekers were also asked how often they seek online health information. However, since these variables were not assessed in 2014 and 2017, they were not analyzed in this article.

The list of the different variables and the corresponding questions asked each year are presented in Multimedia Appendix 1.

#### Ethical Consideration

According to French law, this study was not required to obtain the approval of a national ethics committee, as it is not legally considered research involving human beings and it relied on the collection of anonymous data only.

### Statistical Analysis

Chi-square tests were used to compare the population characteristics over time, including gender, age, educational level, income, employment status, occupational category, and health status (chronic disease). Chi-square tests were also performed to assess the evolution of online health seekers over time and, between 2014 and 2017, of (1) health-related search topics, (2) sources of online health-related information, and (3) types of website used for these searches. The same tests were also used to describe the trends of the trust in health information found online and the potential impact on health management.

Multivariate logistic regression models were performed to investigate the profile of online health seekers versus non–health seekers (defined as the dependent variable), as well as the evolution of the use of the internet for seeking health information over time. Independent variables included in the model were time, all sociodemographic variables, and health status. Interactions between all independent variables and time were assessed to evaluate potential differences in the profile of health seekers over time.

The same models including the same independent variables were performed to evaluate how individuals’ characteristics and time are related with (1) the fact of not paying attention to the source of the health information found online, (2) trust in the information found online, and (3) the change in taking care of one’s health.

Data were weighted by the number of telephone lines and eligible persons in the household. They were also adjusted to represent the French population structure (labor force survey 2008, 2012, and 2014) according to age, gender, educational level, region of residence, and level of urbanization [39].

Given that the maximum age limit was fixed at 75 years in 2014 and 2017 and the minimum at 18 years in 2017, participants aged 15 to 17 years in the 2010 and 2014 surveys and those aged 76 to 85 years in the 2010 survey were excluded in order to allow comparison over time. Given the low rate of missing values among the independent variables (ie, 1.3%), no specific imputation method was employed. Participants were therefore excluded if they had at least one missing value among the covariates used in the models. All tests of statistical significance were 2-sided, and the type I error was set at 5%. Statistical analyses were performed using Stata software version 13 (StataCorp LLC).

## Results

The final sample comprised 15,277 individuals across the 3 time points, respectively 4141 in 2010, 4811 in 2014, and 6255 in 2017. A total of 581 participants were excluded because they were aged younger than 18 years or older than 75 years, and 202 because they had missing data.

Table 1 shows sociodemographic and economic characteristics and health status of included participants over time. Significant differences were found for age, educational level, income, occupational category, and chronic disease. Overall, figures showed that individuals tended to have higher educational levels and household incomes and more chronic diseases over time.

**Figure 1:**
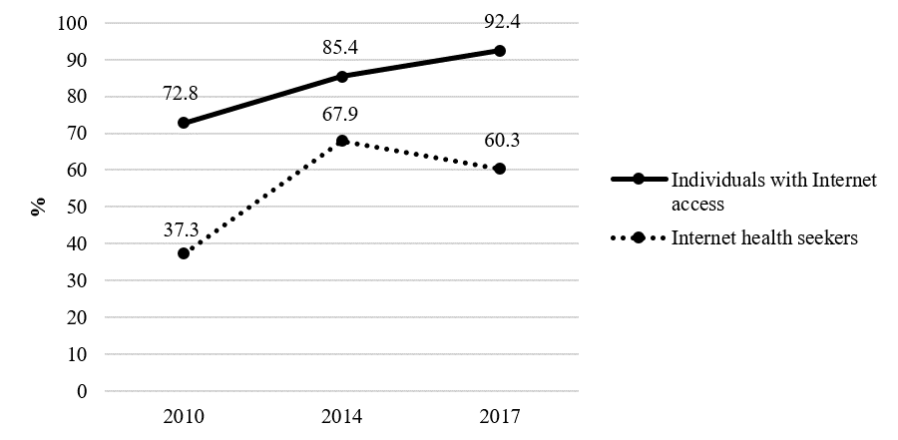
Evolution in internet access and internet use as a source of health information from 2010 to 2017 (2010, N=4141; 2014, N=4881; 2017, N=6255).

The evolution in internet access and use as a source of health information from 2010 to 2017 are presented in Figure 1. Although internet access increased steadily during this period, from 72.8% in 2010 to 92.4% in 2017 (an increase of 27%), the use of the internet as a source of health information rose between 2010 and 2014 (from 37.3% to 67.9%, *P*<.001) and decreased significantly in 2017 (60.3%, *P*<.001).

**Table 1:** Sociodemographic and economic characteristics of included participants over time (2010, 2014, and 2017; N=15,277).

Characteristic		Survey year 2010 (N=4141), n (%)^a^	Survey year 2014 (N=4881), n (%)^a^	Survey year 2017 (N=6255), n (%)^a^	*P* value^b^
**Gender**		—^c^	—	—	.91
	Men	1814 (48.36)	2268 (48.74)	2831 (48.88)	—
	Women	2327 (51.64)	2613 (51.26)	3424 (51.12)	—
**Age in years**		—	—	—	—
	18-24	433 (12.08)	460 (10.62)	503 (10.48)	—
	25-34	745 (17.61)	781 (16.12)	943 (17.28)	—
	35-44	843 (20.02)	1006 (20.54)	1097 (18.55)	—
	45-54	755 (19.65)	1015 (20.46)	1298 (19.63)	—
	55-64	836 (17.68)	923 (18.42)	1308 (18.40)	—
	65-75	529 (12.96)	696 (13.84)	1106 (15.68)	—
**Educational level**		—	—	—	<.001
	Primary	1963 (58.01)	1994 (53.62)	2385 (48.68)	—
	Secondary	791 (18.29)	980 (19.39)	1320 (20.12)	—
	Post-secondary	1387 (23.70)	1907 (27.00)	2550 (31.20)	—
**Income (€/CU^d^)**		—	—	—	<.001
	0-1100	994 (34.89)	1109 (33.47)	1825 (30.76)	—
	1101-1799	1264 (31.25)	1660 (28.83)	2042 (30.62)	—
	≥1800	1606 (25.92)	1854 (31.63)	2106 (33.30)	—
	Not willing to answer	277 (7.94)	258 (6.07)	282 (5.32)	—
**Employment status**		—	—	—	.53
	Working	2440 (57.24)	2989 (57.66)	3619 (56.62)	—
	Student	243 (6.60)	252 (5.57)	332 (6.60)	—
	Unemployed	307 (8.41)	371 (9.56)	442 (8.68)	—
	Retired	915 (20.96)	1035 (20.21)	1503 (21.16)	—
	Other	236 (6.79)	234 (7.00)	359 (6.94)	—
**Occupational category**		—	—	—	.04
	Farmers	70 (1.71)	71 (1.43)	116 (1.82)	—
	Artisans	237 (6.15)	292 (6.50)	379 (6.96)	—
	Executives	759 (14.99)	972 (14.59)	1181 (14.68)	—
	Intermediate profession	1203 (25.37)	1244 (21.61)	1716 (23.69)	—
	Employees	1106 (28.11)	1367 (30.60)	1727 (29.13)	—
	Manual workers	766 (23.68)	935 (25.25)	1136 (23.72)	—
**Chronic disease**		—	—	—	<.001
	No	3138 (76.28)	3135 (65.36)	3948 (64.50)	—
	Yes	1003 (23.72)	1746 (34.64)	2307 (35.50)	—

^a^Percentages are adjusted to represent the French population structure.

^b^On the basis of chi-square tests.

^c^Not applicable.

^d^CU: Household consumer units. One CU is attributed for the first adult in the household, 0.5 for other persons aged 14 years or older and 0.3 for children under 14 years.

Characteristics of online health information seekers across the 3 time points are presented in Table 2. Overall, health information seekers compared with nonseekers were more likely to be women, to be younger, to have a higher educational level, to have a higher household income, and to be executives. As regards the employment status, students and unemployed people were more likely to be health information seekers compared with the working group. Finally, individuals having a chronic disease were more likely to be health information seekers. Significant interaction with time was observed for age, educational level, and income. Results by the year of the survey were therefore explored (see Multimedia Appendix 2). Over time, the gap between generations seemed to be widening. In 2010, no differences in the use of the internet for seeking health information were found with people aged 45 to 54 years and younger, whereas this was the case in 2014 and 2017. As regards educational level, trends were comparable over time, but the odds of being online health seekers varied, in particular among those with a secondary education level. Finally, income was found to be less predictive of online seeking behavior after 2010, and particularly in 2014, since there was no more significant difference between the lower and intermediate income levels.

**Table 2:** Multivariate regression logistic models showing the association of internet use for seeking health information with time and individual characteristics (2010, N=4141; 2014, N=4881; 2017, N=6255).

Characteristic			OR^a^ (95% CI)		*P* value^b^		*P* value of the time interaction^c^
**Year**			—^d^		—		—
	2010	1		—		—	
	2014	4.23 (3.76-4.75)		<.001		—	
	2017	2.71 (2.43-3.02)		<.001		—	
**Gender**			—		—		.14
	Men	1		—		—	
	Women	1.81 (1.64-1.99)		<.001		—	
**Age in years**			—		—		.0001
	18-24	1		—		—	
	25-34	1.30 (1.05-1.61)		.02		—	
	35-44	0.90 (0.73-1.12)		.35		—	
	45-54	0.57 (0.46-0.70)		<.001		—	
	55-64	0.45 (0.36-0.57)		<.001		—	
	65-75	0.26 (0.19-0.34)		<.001		—	
**Educational level**			—		—		.02
	Primary	1		—		—	
	Secondary	1.62 (1.44-1.82)		<.001		—	
	Up to secondary	2.13 (1.89-2.41)		<.001		—	
**Income (€/CU^e^)**			—		—		.03
	1-1100	1		—		—	
	1101-1799	1.26 (1.12-1.42)		<.001		—	
	≥1800	1.56 (1.37-1.78)		<.001		—	
	Not willing to answer	0.74 (0.60-0.91)		.004		—	
**Employment status**			—		—		.49
	Working	1		—		—	
	Student	1.67 (1.28-2.17)		<.001		—	
	Unemployed	1.22 (1.02-1.45)		.03		—	
	Retired	0.90 (0.74-1.09)		.27		—	
	Other	0.90 (0.73-1.12)		.35		—	
**Occupational category**			—		—		.57
	Executive	1		—		—	
	Intermediate profession	0.85 (0.74-0.98)		.03		—	
	Employee	0.70 (0.60-0.82)		<.001		—	
	Artisan	0.71 (0.57-0.88)		.002		—	
	Manual worker	0.52 (0.44-0.62)		<.001		—	
	Farmer	0.43 (0.30-0.61)		<.001		—	
**Chronic disease**			—		—		.76
	No	1		—		—	
	Yes	1.58 (1.43-1.75)		<.001		—	

^a^OR: odds ratio.

^b^Multivariate logistic regression adjusted for year, gender, age, educational level, household income, employment status, occupational category, and chronic disease.

^c^*P* value of the interaction term when adding interaction between each variable and year of survey in the logistic model.

^d^Not applicable.

^e^CU: consumption unit. One CU is attributed for the first adult in the household, 0.5 for other persons aged 14 years or older and 0.3 for children under 14 years.

Table 3 shows the topics of online health information research in 2014 and 2017, as well as the online sources of information and the type of websites used. Overall, the main topics searched online remained the same in 2014 and 2017 (ie, general health and illnesses; medical news and treatments; nutrition, weight gain, or eating disorders; and child health and illness). Nonetheless, while the percentage of respondents searching for information about general health and child health decreased over time, the percentage concerning nutrition-related topics remained constant. The most significant decreases were observed for searches about tobacco (–41%), alcohol (–70%), and cannabis and other drugs (–38%).

Overall, while the proportion of people using known health information websites and forums decreased, the proportion of those who did not pay attention to the source significantly increased (+12.1 percentage points). Thus, in 2017, this was the case for about 4 out of 10 internet health information seekers.

When focusing on the type of websites used for the last health-related internet searches by individuals who paid attention to the information source (48.7% in 2014 and 46.8% in 2017), figures indicates that between 2014 and 2017 general health-related websites remained the main source of information. Social media and commercial websites were the second source of information in 2014 as in 2017. And, in 2017, institutional websites were the third source. However, even if the visits to these institutional websites increased between 2014 and 2017, they remained at relatively low level, with only 8.1% of individuals declaring they used these sources.

**Figure 2:**
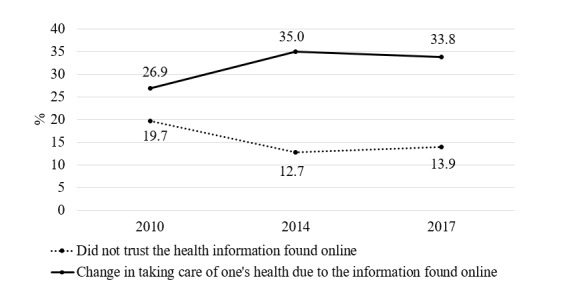
Evolution of the trust in health information found on the internet and the change in taking care of one’s health from 2010 to 2017 (2010, N=1707; 2014, N=3582; 2017, N=3965).

Figure 2 shows the evolution of the trust in health information found on the internet and the change in taking care of one’s health. Globally, the evolutions were symmetrical. While trust in the information increased from 2010 and 2014 (*P*<.001) and decreased slightly from 2014 to 2017 (*P*=.048), the change in health management rose between 2010 and 2014 (*P*<.001) but remained stable between 2014 and 2017. Nonetheless, the perception of health-related information found online was relatively positive, with at least 80% reporting that the most recent information found was trustworthy.

**Table 3:** Evolution between 2014 and 2017 among internet health seekers of (1) health-related search topics, (2) sources of online health-related information, and (3) types of websites used for internet health-related searches.

Search topics			Survey year 2014, n (%)^a^		Survey year 2017, n (%)^a^		*P* value^b^
**(1) Health-related search topics**			n=2036		n=3917		
	General health and illnesses, medical news, and treatments^c^	1022 (71.80)		2159 (64.72)		.001	
	Nutrition, weight gain, or eating disorders	921 (45.03)		1741 (44.96)		.23	
	Child health and illness	625 (33.13)		975 (27.16)		<.001	
	Pregnancy or maternity	268 (14.63)		388 (11.78)		.02	
	Tobacco	240 (12.40)		245 (7.30)		<.001	
	Electronic cigarette	—^d^		191 (4.97)		—	
	Alcohol	197 (11.54)		133 (3.39)		<.001	
	Contraception and method to avoid pregnancy	164 (9.60)		237 (7.38)		.047	
	Sexually-related health risk	131 (7.41)		211 (6.59)		.37	
	Cannabis and other drugs	137 (7.12)		155 (4.36)		.001	
**(2) Sources of online health-related information**			n=1396		n=3917		
	Health information website	816 (55.75)		2035 (48.83)		<.001	
	Forum	432 (32.38)		801 (21.55)		<.001	
	active on the forum (n=1233)	20 (4.41)		40 (5.44)		.48	
	Did not pay attention to the source of the information	370 (27.63)		1475 (39.74)		<.001	
**(3) Type of websites used for internet health-related searches (among those who consulted information websites)**			n=1478		n=2442		
	General health-related website	443 (30.25)		937 (38.58)		<.001	
	Doctissimo^e^	371 (25.43)		733 (29.99)		.009	
	Social medias (YouTube, Facebook, blogs, TV...) and commercial websites	209 (13.70)		285 (11.86)		.16	
	Wikipedia	94 (6.16)		87 (3.16)		<.001	
	Institutional websites	103 (5.98)		212 (8.08)		.02	
	Websites from health professional, patient association, scientific database	66 (3.86)		161 (6.01)		.006	
	Other	18 (1.14)		6 (0.16)		.002	

^a^Percentages are adjusted to represent the French population structure.

^b^On the basis of chi-square tests.

^c^For the “general health and illnesses, medical news, and treatments” topic, the question was asked to a subsample of participants to the study (n=4607).

^d^Not applicable.

^e^A popular French website dedicated to general health mentioned by name by a large number of participants.

The results of multivariate models showing how individuals’ characteristics and time are related to (1) the fact of not paying attention to the source of the health information found online, (2) trust in the information found online, and (3) the change in taking care of one’s health are shown in Table 4. Not paying attention to the health-related information source significantly increased between 2014 and 2017. Such practice was associated with a higher probability of having lower education levels and being artisans or manual workers. In turn, students were more likely to pay attention to the source of their searches.

Trust in the health information found online and change in taking care of one’s health both significantly increased in 2014 and slightly decreased in 2017. Regarding the associations with sociodemographic characteristics, only individuals with higher educational levels were more likely to trust the information found online. This trust strongly influenced the way of managing one’s health (odds ratio 4.07). Individuals aged 25 to 34 years were more likely to have changed the way they managed their health due to the information found online, whereas those aged 65 to 75 years were less likely to do so. Finally, those having higher educational levels and higher incomes were also less influenced by the information found online.

**Table 4:** Multivariate logistic regression models showing the association of individuals’ characteristics and time with (1) the fact of not paying attention to the source of the health information found online (2014, N=1422; 2017, N=3965), (2) trust in the information found online, and (3) the change in taking care of one’s health (both 2010, N=1707; 2014, N=3582; 2017, N=3965).

Characteristic		Not paying attention to information source (N=5313), OR^a^ (95% CI)	*P* value^b^	Trust in the last health information found online, OR (95% CI)	*P* value^b^	Change in taking care of one’s health, OR (95% CI)	*P* value^b^
**Year**							
	2010	—^c^	—	1	—	1	—
	2014	1	—	1.66 (1.35-2.04)	<.001	1.41 (1.19-1.65)	<.001
	2017	1.78 (1.50-2.11)	<.001	1.53 (1.27-1.85)	<.001	1.32 (1.13-1.55)	<.001
**Trust in health information found online**							
	Not trustworthy	—	—	—	—	1	—
	Trustworthy	—	—	—	—	4.06 (3.26-5.06)	<.001
**Gender**							
	Men	1	—	1	—	1	—
	Women	1.02 (0.87-1.19)	.80	0.93 (0.78-1.10)	.40	0.89 (0.79-1.01)	.07
**Age in years**							
	18-24	1	—	1	—	1	—
	25-34	0.96 (0.68-1.34)	.79	0.99 (0.72-1.37)	.96	1.37 (1.05-1.78)	.02
	35-44	0.93 (0.66-1.32)	.69	1.32 (0.94-1.85)	.11	1.20 (0.92-1.58)	.18
	45-54	1.12 (0.79-1.59)	.53	1.31 (0.92-1.85)	.13	1.21 (0.92-1.60)	.17
	55-64	1.27 (0.87-1.85)	.21	1.10 (0.74-1.64)	.63	0.77 (0.57-1.05)	.11
	65-75	1.39 (0.86-2.25)	.18	0.91 (0.55-1.51)	.72	0.70 (0.46-1.05)	.08
**Educational level**							
	Primary	1	—	1	—	1	—
	Secondary	0.66 (0.54-0.80)	<.001	1.09 (0.88-1.33)	.43	0.86 (0.73-1.01)	.06
	Up to secondary	0.57 (0.47-0.70)	<.001	1.31 (1.05-1.63)	.03	0.87 (0.74-1.02)	.08
**Income (€/CU^d^)**							
	0-1100	1	—	1	—	1	—
	1101-1799	0.90 (0.74-1.10)	.31	1.15 (0.93-1.43)	.19	0.82 (0.69-0.96)	.01
	≥1800	0.91 (0.74-1.13)	.39	1.28 (1.02-1.62)	.04	0.79 (0.67-0.94)	.007
	Not willing to answer	1.05 (0.71-1.55)	.81	0.91 (0.61-1.36)	.65	1.13 (0.84-1.53)	.42
**Employment status**							
	Working	1	—	1	—	1	—
	Student	0.65 (0.44-0.96)	.03	1.31 (0.90-1.90)	.16	1.25 (0.93-1.67)	.14
	Unemployed	0.95 (0.72-1.25)	.71	1.21 (0.88-1.65)	.25	1.54 (1.23-1.92)	<.001
	Retired	0.91 (0.66-1.25)	.56	1.32 (0.92-1.90)	.13	1.33 (1.02-1.73)	.04
	Other	0.87 (0.62-1.22)	.43	1.02 (0.71-1.47)	.91	1.58 (1.20-2.09)	.001
**Occupational category**							
	Executive	1	—	1	—	1	—
	Intermediate profession	1.05 (0.85-1.29)	.66	1.04 (0.84-1.28)	.73	1.54 (0.83-2.84)	.17
	Employee	1.19 (0.94-1.51)	.15	1.14 (0.88-1.47)	.33	1.31 (0.73-2.35)	.36
	Artisan	1.91 (1.35-2.71)	<.001	1.39 (0.90-2.14)	.14	1.41 (0.79-2.50)	.25
	Manual worker	1.48 (1.13-1.95)	.005	0.95 (0.71-1.27)	.75	1.52 (0.85-2.70)	.16
	Farmer	1.28 (0.65-2.52)	.47	0.78 (0.38-1.58)	.49	1.60 (0.89-2.87)	.12
**Chronic disease**							
	No	1	—	1	—	1	—
	Yes	0.46 (0.30-0.72)	.001	1.08 (0.91-1.28)	.40	0.97 (0.85-1.10)	.63

^a^OR: odds ratio.

^b^Multivariate logistic regression adjusted for year, gender, age, educational level, household income, employment status, occupational category, and chronic disease.

^c^Not applicable.

^d^CU: consumption unit. One CU is attributed for the first adult in the household, 0.5 for other persons aged 14 years or older, and 0.3 for children under 14 years.

## Discussion

### Principal Findings and Interpretation

This was one of the first studies describing the evolution of online health information seeking in a European country, based on nationally representative time-series survey data. We observed an increase of the use of the internet for health-related information between 2010 and 2014 but a decrease between 2014 and 2017. In parallel, trust in the health information found online followed the same trend, thus suggesting a potential relationship between these 2 variables. Indeed, the growing phenomenon of misinformation and fake news might restrain citizens from using the internet for health-related information [40]. They might prefer consulting a health professional or just avoid looking for health information online [41]. However, promoting access to trustworthy information online represents a key lever to help people managing their health. Therefore, a growing body of research exists on the potential of interventions designed to develop eHealth literacy [42], which has been described as a necessary competence to mitigate health inequalities [43].

The rise in distrust was paradoxically complemented by a higher proportion of respondents reporting not paying attention to the information sources. This might be explained by the fact that it is often difficult to identify the source of information and assess its credibility. Apart from institutional health-related websites (eg, the website of the Ministry of Health, the website of a local hospital), determining the online source of information has become challenging and even frustrating [44]. On the other hand, general websites are easy to access and consult, while institutional websites remain less consulted, even if a slight increase was reported between 2014 and 2017 in our study. The complexity and density of the information they provide might explain their scarce use, despite their trustworthiness. Those who trusted more online contents were respondents having higher educational levels and incomes, which might be explained by the fact that they are supposed to have more developed eHealth literacy and technological skills, allowing them to better evaluate the accuracy of the information retrieved online [13,45]. These citizens were also less likely to change the management of their health based on health-related information found online, differentiating their information-seeking behavior from their health behavior [46].

Sociodemographic characteristics and health conditions of online health information seekers were similar to those found in previous studies [14,18,47]; being a woman, being younger, being an executive, having a higher educational level, having a higher household income, and having a chronic disease were all associated with use of the internet for health information seeking. Interestingly, our results showed that unemployed people were more likely to be health information seekers compared with other groups such as working or retired people. This result is in contrast with previous research [48,49] but in line with other studies [19,50] showing that this point is controversial and might depend on the specific characteristics of unemployed citizens when they are not taken into account in a model, like the fact of being a woman or having an illness, or rather on the country’s unemployment rate or medical care coverage for these people. What can be said with more confidence is that higher educational levels are associated with higher use of the internet for health information seeking, independently from being employed or not, since eHealth literacy skills are higher among people having a diploma [51]. In our study, students, independently of the level of education, were more likely to be health information seekers compared with other people (eg, working, retired), which can be explained by the fact that they are used to seeking online information in general as part of their study curriculum.

Trends about searched topics were similar across time periods, with general health and illness being the most searched terms together with nutrition, weight gain, or eating disorders and child health and illness. The prevalence of these topics is explained by the characteristics of likely online health information seekers (ie, women aged 35 to 54 years). Gender differences have been frequently reported as relevant for health information seeking, including topics of interest [52]. Decreasing interest in topics like tobacco, alcohol, or other drug consumption might be explained by the fact that users prefer browsing the web for general health-related information, while for more specific problems like addiction, they prefer other sources of information. This is in line with the trustworthiness of online information and the risk of encountering fake news for sensitive topics like drug consumption and is also documented in previous research [53].

### Strengths and Limitations

Strengths of this study included the use of large datasets from nationally representative surveys including the general population with various sociodemographic characteristics. The time-series design was also important to robustly assess the evolution of online health information-seeking patterns in the French population.

This study is not without limitations. First, while being based on large samples, the response rates were between 48.5% and 61%, which means that selection bias cannot be excluded and that some specific population groups like homeless people or immigrants were likely to be underrepresented. Second, as a population-based study specific to France, these results are not generalizable to other countries, although online health information-seeking behavior patterns are supposed to be similar in most of the European countries. Third, data on trust in the information found online were only available for the subsample of health information seekers: this prevented the evaluation of the association between trust and decrease in the use of the internet for health information seeking. Fourth, Health Barometer surveys do not report on important aspects related to online health information seeking such as technical skills and eHealth literacy. Finally, the reliability of some answers may be affected by a memory bias, and other data concerning online health information seeking were not assessed like the frequency of use and the use of social media or mobile apps providing health tips and information. A complete picture of online health information-seeking behaviors might benefit from more data on digital health use in general.

### Conclusions

Our results showed a rapid growth in internet use in the 2010 to 2014 period with a decrease in the year 2017, in parallel with a decreasing trust in the quality and reliability of information found online. The trends in the use of and trust in online health information need to be constantly monitored, but our findings already underlined the need for alternative trustworthy sources of information on the internet. In particular, it is recommended that official health institutions promote initiatives to help citizens navigate health-related information available on the internet. These initiatives might range from interventions aimed at promoting citizens’ eHealth literacy, such as educational programs, to official websites and online portals providing reliable but simple and usable information. Effective interventions should combine the popularity and accessibility of general health-related websites with the authority of the institutional websites.

## Appendix

Multimedia Appendix 1 List of the variables used in the study and the corresponding questions asked by year of survey.

Multimedia Appendix 2 Multivariate regression logistic models showing the association of internet use for seeking health information with individual characteristics by the year of the survey (2010, N=4141; 2014, N=4881; 2017, N=6255).

## Abbreviations

CU: consumption unit
INPES: French Institute for Prevention and Health Education
