# Supplementary material for: Evolution of Online Health-Related Information Seeking in France From 2010 to 2017: Results From Nationally Representative Surveys
Source: J Med Internet Res. 2021 Apr 14;23(4):e18799. doi: 10.2196/18799 (PMC8082381; doi:10.2196/18799)
Supplement: Multimedia Appendix 1 [file jmir_v23i4e18799_app1.docx]

Supplemental Table 1. List of the variables used in the study and the corresponding question asked by year of survey

| **Variables** | **Questions** | **Year of the survey** | | |
| --- | --- | --- | --- | --- |
|  |  | **2010** | **2014** | **2017** |
| **Internet use** | Do you ever use the Internet? | x | x | x |
| **Internet use for seeking health-information** | During the past twelve months, have you used internet to seek information about health? | X | X | X |
| **Health-related search topics** | Last time, on what subjects did you consult health information on the Internet? | - | subsample 1 | X |
| **Source of health information** | Last time you looked for health information on the Internet, was it …on a forum / an information website / you did not pay attention | - | subsample 1 | X |
| **Types of website used for health-related Internet searches *(among those who consulted information websites)*** | Last time, what sites did you go to for health information on the internet? | - | subsample 2 | X |
| **Change in taking care of one's health** | Overall, have the information and advice you found on the Internet changed the way you look after your health? | X | X | X |
| **Trust in the last health information found online** | In your opinion, is the health information you obtained last time trustworthy? | X | X | X |
