# Supplementary material for: Evolution of Online Health-Related Information Seeking in France From 2010 to 2017: Results From Nationally Representative Surveys
Source: J Med Internet Res. 2021 Apr 14;23(4):e18799. doi: 10.2196/18799 (PMC8082381; doi:10.2196/18799)
Supplement: Multimedia Appendix 2 [file jmir_v23i4e18799_app2.docx]

Supplemental Table 2. Multivariate regression logistic models showing the association of Internet use for seeking health information with individual characteristics by the year of the survey (2010, N=4,141-2014, N=4,881-2017, N=6,255)

|  | **2010** | **2014** | **2017** |
| --- | --- | --- | --- |
|  | **N=4,141** | **N=4,881** | **N=6,255** |
|  | **OR [95%CI]** | **OR [95%CI]** | **OR [95%CI]** |
| **Gender** |  |  |  |
| Men | 1 | 1 | 1 |
| Women | 1.67 [1.40;1.99]*** | 1.77 [1.47;2.14]*** | 1.96 [1.69;2.27]*** |
| **Age** |  |  |  |
| 18-24 | 1 | 1 | 1 |
| 25-34 | 1.19 [0.83;1.70] | 1.40 [0.91;2.16] | 1.35 [0.93;1.95] |
| 35-44 | 1.10 [0.76;1.58] | 0.72 [0.47;1.09] | 0.87 [0.61;1.24] |
| 45-54 | 0.84 [0.58;1.22] | 0.42 [0.28;0.62]*** | 0.51 [0.36;0.72]*** |
| 55-64 | 0.62 [0.41;0.93]* | 0.34 [0.22;0.53]*** | 0.43 [0.30;0.62]*** |
| 65-75 | 0.26 [0.15;0.44]*** | 0.15 [0.09;0.26]*** | 0.34 [0.22;0.54]*** |
| **Educational level** |  |  |  |
| Primary | 1 | 1 | 1 |
| Secondary | 1.67 [1.34;2.07]*** | 2.06 [1.64;2.59]*** | 1.38 [1.15;1.66]*** |
| Post-secondary | 2.47 [1.98;3.07]*** | 2.01 [1.59;2.53]*** | 2.03 [1.68;2.45]*** |
| **Income (€/CU^b^)** |  |  |  |
| 0-1100 | 1 | 1 | 1 |
| 1101-1799 | 1.53 [1.24;1.89]*** | 0.93 [0.74;1.17] | 1.33 [1.10;1.60]** |
| ≥1800 | 1.81 [1.43;2.29]*** | 1.41 [1.10;1.82]** | 1.49 [1.22;1.83]*** |
| Not willing to answer | 0.87 [0.61;1.24] | 0.62 [0.42;0.90]* | 0.73 [0.52;1.02] |
| **Employment status** |  |  |  |
| Working | 1 | 1 | 1 |
| Student | 2.06 [1.32;3.21]** | 1.05 [0.63;1.76] | 1.85 [1.20;2.86]** |
| Unemployed | 1.26 [0.92;1.73] | 1.02 [0.72;1.44] | 1.39 [1.06;1.83]* |
| Retired | 0.92 [0.65;1.30] | 0.94 [0.66;1.35] | 0.82 [0.62;1.07] |
| Other | 1.02 [0.70;1.48] | 0.76 [0.50;1.16] | 0.96 [0.70;1.31] |
| **Occupational category** |  |  |  |
| Executives | 1 | 1 | 1 |
| Intermediate profession | 1.03 [0.81;1.30] | 0.71 [0.53;0.96]* | 0.83 [0.67;1.03] |
| Employees | 0.83 [0.62;1.10] | 0.54 [0.39;0.74]*** | 0.74 [0.58;0.95]* |
| Artisans | 0.68 [0.45;1.02] | 0.70 [0.46;1.07] | 0.75 [0.54;1.05] |
| Manual workers | 0.62 [0.46;0.85]** | 0.42 [0.30;0.58]*** | 0.54 [0.42;0.70]*** |
| Farmers | 0.50 [0.25;1.01] | 0.38 [0.20;0.72]** | 0.42 [0.24;0.72]** |
| **Chronic disease** |  |  |  |
| **No** | 1 | 1 | 1 |
| **Yes** | 1.51 [1.24;1.84] | 1.81 [1.49;2.19] | 1.48 [1.28;1.71] |

*** *P*<.001 ; ** *P*<.01 ; * P<.05
